# Cognitive remediation for depression vulnerability: Current challenges and new directions

**DOI:** 10.3389/fpsyg.2022.903446

**Published:** 2022-07-22

**Authors:** Yannick Vander Zwalmen, Kristof Hoorelbeke, Eveline Liebaert, Constance Nève de Mévergnies, Ernst H. W. Koster

**Affiliations:** ^1^Department of Experimental Clinical and Health Psychology, Ghent University, Ghent, Belgium; ^2^Department of Head and Skin, Ghent University Hospital, Ghent, Belgium

**Keywords:** cognitive control training, depression, working memory training, cognitive remediation, prevention of depression

## Abstract

It is increasingly acknowledged that cognitive impairment can play an important role in depression vulnerability. Therefore, cognitive remediation strategies, and cognitive control training (CCT) procedures have gained attention in recent years as possible interventions for depression. Recent studies suggest a small to medium effect on indicators of depression vulnerability. Despite initial evidence for the efficacy and effectiveness of CCT, several central questions remain. In this paper we consider the key challenges for the clinical implementation of CCT, including exploration of (1) potential working mechanisms and related to this, moderators of training effects, (2) necessary conditions under which CCT could be optimally administered, such as dose requirements and training schedules, and (3) how CCT could interact with or augment existing treatments of depression. Revisiting the CCT literature, we also reflect upon the possibilities to evolve toward a stratified medicine approach, in which individual differences could be taken into account and used to optimize prevention of depression.

Depression is a highly prevalent psychiatric disorder, affecting millions of people worldwide (World Health Organization [WHO], 2021). One of the core aspects of depression is impaired cognitive control (i.e., the ability to flexibly adapt thoughts and behavior in order to achieve one’s goals; Cohen, 2017), which is known to persist even after recovery (Semkovska et al., 2019). Cognitive control impairments are typically not directly targeted by antidepressant treatments (Shilyansky et al., 2016). Importantly, there is increasing evidence suggesting that cognitive control impairments play a key role in risk for (re)occurrence of depression (Buckman et al., 2018). For instance, Vanderhasselt and de Raedt (2009) found that deficits in cognitive control increase with each depressive episode and persist after symptom remission. In addition, Demeyer et al. (2012) found that impaired cognitive control at baseline was predictive of depressive symptomatology at 1 year follow-up in a remitted depressed sample (RMD). Furthermore, cognitive impairments are an important source of disability in daily life following remission from depression (Knight et al., 2018). Therefore, recent years have seen increased interest in the use of novel approaches to remediate these neurocognitive impairments. One such intervention, previously employed for other psychiatric disorders, is cognitive control training (CCT), in which working memory and executive functioning are trained. CCT has been operationalized in different ways, often using adaptive versions of working memory paradigms, such as the Eriksen Flanker task (Eriksen and Eriksen, 1974), the n-back (Kirchner, 1958) or the Paced Auditory Serial Addition Task (PASAT; Gronwall, 1977). Recent studies examining the influence of motivation on CCT found that by adding elements of gamification, training motivation and treatment adherence could be increased, without lowering its effectiveness (e.g., n-back: Mohammed et al., 2017; PASAT: Vervaeke et al., 2020).

In this paper we discuss the current state-of-the-art of CCT as an intervention to reduce depression vulnerability. Initial findings have suggested that this could be a useful intervention to reduce depression vulnerability (Siegle et al., 2007, 2014; Calkins et al., 2015) but challenges in several domains remain to further develop CCT into a successful clinical intervention. Based on the current state of the literature, the key challenges that we discuss are (i) identifying working mechanisms and potential moderators of CCT; (ii) establishing conditions for effectively applying CCT; (iii) evaluating possible combinations of CCT with other antidepressant interventions.

## Working mechanisms

The working mechanisms of CCT are currently not completely understood. One possible theory highlights the importance of impaired attentional disengagement in depression (Koster et al., 2011). When experiencing stress, negative self-referent thoughts and feelings can arise. Although most people are able to manage negative thoughts, sometimes people get stuck in this process, and fail to disconnect from their negative thinking, resulting in a loop of negative self-referential thinking. Repetitive negative thinking (RNT) and rumination have been frequently associated with, and are a core part of, depression (Nolen-Hoeksema et al., 2008). CCT is believed to beneficially impact attentional disengagement from self-referential negative thoughts and feelings, allowing one to break free from RNT and its possible effects on depressed mood (Koster et al., 2011). Another model illustrating the relationship between depressive rumination and cognitive control, the H-EX-A-GO-N model from Watkins and Roberts (2020), describes the influence of habitual interactions with other factors in the development of rumination. Here, executive functioning impairments weaken one’s ability to disengage from habitual ruminative responses, thereby increasing the likelihood of employing ruminative behavior as a habitual response to negative mood. The formation of ruminative habits, paired with executive functioning impairments, could help explain why people experience trouble interrupting ruminative processes, even when they are aware of its unhelpfulness. Indeed, habits can be resistant and difficult to change, even when they are conflicting with one’s goals or intentions. Both the attentional disengagement (Koster et al., 2011) and the hexagon model (Watkins and Roberts, 2020) highlight the importance of cognitive control for RNT. By improving cognitive control, cognitive and behavioral decoupling from depressive rumination could be a possible working mechanism through which CCT could influence depression vulnerability.

Another perspective, the capacity-efficiency framework, focuses on the role of transfer effects of CCT (for a review, please see von Bastian et al., 2022). In this framework, cognitive training can expand cognitive capacity, or it can increase the efficiency of the already present cognitive abilities. These two possible working mechanisms are not mutually exclusive: it is possible that both cognitive capacity increases *and* efficiency increases simultaneously. In the context of CCT interventions targeting depression vulnerability, it is unclear to what extent existing training procedures target capacity, efficiency, or both. This is likely to be impacted by the nature of the training task and the adaptive mechanism underlying the training procedure. Increased cognitive processing efficiency may be reflected in increased performance on tasks which more strongly load on working memory capacity (e.g., working memory span tasks).

Essential in this framework is that potential cognitive gains do not stem from learning specific strategies or general task familiarity. Instead, by training cognitive control the effects transfer to other domains of cognitive or emotional functioning. These transfer effects are thus assumed not to be task-dependent and reflect real cognitive gains. Evidence for near transfer (i.e., transfer to tasks relying on the same underlying cognitive construct, such as working memory) and far transfer (i.e., transfer to different cognitive abilities) has been found in CCT studies, but prior research has yielded inconsistent results, with near transfer more frequently being observed than far transfer (Sala et al., 2019). It has been suggested for future CCT studies to include non-adaptive tasks, so that both near and far transfer can be examined (Koster et al., 2017). In this context, investigating how training progress and cognitive transfer relate to patterns of emotional transfer may shed light on the mechanisms underlying effects of CCT. For instance, Hoorelbeke and Koster (2017) observed task-specific cognitive transfer following CCT to be predictive of change in residual depressive symptomatology in remitted depressed individuals. This effect was partially mediated by immediate effects of CCT on RNT, suggesting that one potential mechanism through which CCT may impact risk for depression may indeed be increased disengagement from negative self-referential thoughts.

In addition to studies investigating effects of CCT on cognitive task performance and measures of emotional transfer, numerous studies have explored neuropsychological effects of CCT. Research has indicated that CCT activates the dorsolateral prefrontal cortex (DLPFC; Siegle et al., 2007). The DLPFC is known to be implicated in executive functioning and plays a central role in maintaining, discarding, and manipulating information in working memory (Barbey et al., 2013). The prefrontal and limbic regions are often activated together and play an important role in the relationship between cognition and emotion. It is hypothesized that the DLPFC is involved in regulating emotions and by improving cognitive control, one might strengthen emotion regulation capabilities. Some studies found that, even after recovery from depression, RMD individuals still show lower levels of DLPFC activation (Hooley et al., 2009). In times of distress, when amygdala activation is high, prefrontal cortical control is required to regulate limbic activity. Indeed, impaired cognitive control and decreased prefrontal activity in (remitted) depressed individuals might in part explain the high recurrence rates. In this context, initial studies suggest CCT to beneficially impact patterns of dysfunctional activation in frontal and limbic regions in patients undergoing treatment for major depressive disorder (MDD; Siegle et al., 2007). Similarly, in a convenience sample, Cohen et al. (2016) found CCT to increase connectivity between the amygdala and prefrontal regions. Such findings allow to link observed patterns of cognitive and emotional transfer, where further investigation into the underlying pathways of training effects is likely to yield possible targets for increasing the efficacy of CCT.

## Treatment efficacy and its moderators

In terms of effectiveness, recent meta-analyses examining CCT for depressive symptomatology have found small to medium effect sizes, indicating that CCT could be a useful intervention in the context of depression (Motter et al., 2016; Launder et al., 2021). Interestingly, these effect sizes are very similar to those of other antidepressant treatments, such as pharmacological interventions (Khan and Brown, 2015) and psychological therapies (Cuijpers et al., 2013), highlighting both the clinical need for new treatment options, as well as warranting the use of CCT in clinical practice. In order to increase its efficacy, more insight into moderators of training effects is required. Previous studies have reported effects of CCT on depression vulnerability in different populations, such as patients diagnosed with MDD (Siegle et al., 2014), RMD individuals (Hoorelbeke and Koster, 2017), at-risk (Hoorelbeke et al., 2015; Beloe and Derakshan, 2019) and healthy individuals (Vervaeke et al., 2020). It has been posited that, in order to train cognitive control, interindividual differences might be more indicative of treatment response than categorical population type differences, and that individuals with different baseline levels of cognitive functioning might respond differently to cognitive training (Jaeggi et al., 2013; Borella et al., 2017; Traut et al., 2021). Indeed, some studies suggest that people scoring poorly on cognitive tasks could benefit the most (Karbach et al., 2014, 2017). At the same time, it might be the case that a certain level of cognitive control or executive functioning is required to profit from CCT procedures, meaning that the people showing the most cognitive impairment (regardless of their MDD or RMD status) might first need to reinforce their current cognitive capacity before CCT could improve them substantially (Traut et al., 2021). If this is the case, then cognitive training gains may not necessarily be a linear process: people with low cognitive control might first require an increase in cognitive control before training could further improve cognitive control.

Individual differences in baseline (cognitive) functioning may also reflect differential training needs in terms of training dosage. The utilization of baseline differences to differentiate a potential CCT treatment is currently not in widespread use but could potentially be a way in which CCT could be personalized and tailored to the individual. In this context, several studies have explored the association between specific factors, among which baseline level of cognitive functioning (e.g., Moshier and Otto, 2017), training task progress (e.g., Vanderhasselt et al., 2015), training related cognitive gains (e.g., Peckham and Johnson, 2018), level of RNT (e.g., Daches et al., 2015), and CCT efficacy. This has yielded inconsistent findings and leads us to believe that, similar to other anti-depressant interventions, there might not be a “one-size-fits-all” solution in terms of training operationalization for depression.

In this context, it has been suggested that a more integrative approach may be required in order to better capture profiles of functioning (Hoorelbeke et al., 2022). For instance, in a study examining subgroups based on baseline patient characteristics, interesting profiles emerged for the treatment and prevention of depression (Saunders et al., 2021). Patients with long durations of depression and anxiety, moderate to severe symptoms and past antidepressant use appeared to benefit more from psychotherapy, than antidepressant medication or treatment as usual (TAU). Moreover, profiles with greater risk of poor outcomes appeared to benefit more from more intensive treatment and frequent monitoring. Similar results were found in a study in which latent profile analysis was conducted to identify user profiles based on broad indicators of functioning, among which baseline level of cognitive functioning, use of (mal)adaptive emotion regulation strategies, level of internalizing symptomatology, training progress, user experience, and personality factors (Hoorelbeke et al., 2022). In particular, this study identified three user profiles, reflecting low-, moderate- and high-risk for internalizing psychopathology. These profiles moderated task-specific cognitive transfer effects, as well as change in anxiety- and stress symptoms following CCT. CCT seemed to be more effective for the moderate- and high-risk groups. Moreover, it was suggested that the high-risk group could potentially benefit from an increased training dosage, as shown by relatively short-lived emotional transfer effects compared to the moderate training group, and less extensive cognitive training gains. Employing a stratified medicine approach by administrating CCT specifically for patients whom would benefit from such training is another possible way through which CCT could evolve into a personalized treatment for depression.

## Treatment combinations

Although CCT has shown promise in reducing depression vulnerability, among which level of depressive symptomatology (e.g., Siegle et al., 2007; Hoorelbeke and Koster, 2017), CCT is unlikely to be a sufficiently effective stand-alone treatment for clinical populations. Hence, it is important to consider its combination with other interventions for depression (see also Van den Bergh et al., 2018). Meta-analyses show that combining psychotherapy with anti-depressant medication can result in improved treatment effects compared to monotherapy. However, research examining the antidepressant effects of combining CCT with other interventions is still scarce. One study examined the combination of CCT with the antidepressant vortioxetine (Lenze et al., 2020) and found that combined treatment led to a greater increase in global cognitive performance compared to only CCT (i.e., without antidepressant medication) in a sample of older adults who reported cognitive dysfunctions. The authors concluded that the combined treatment could be beneficial to counteract age-related cognitive decline.

Other studies have evaluated additive effects of CCT when combined with psychotherapeutic interventions. For instance, one study investigated the added value of CCT to a treatment consisting of behavior activation (Moshier and Otto, 2017), whereas another study explored additive effects of CCT using a group-based cognitive behavior therapeutic fear of failure program (Van den Bergh et al., 2020). Both studies failed to find evidence for treatment augmentation when combining CCT with a psychotherapeutic intervention. In contrast, Course-Choi et al. (2017) reported beneficial effects of a combined CCT and mindfulness approach to target RNT.

Another interesting avenue is the combination of CCT with neuromodulation strategies such as transcranial Direct-Current Stimulation (tDCS). It is assumed that tDCS in combination with CCT aims to increase CCT effectiveness, rather than induce effects by itself (Weller et al., 2020). In a study using healthy young adults, anodal tDCS to the left PFC improved performance in an n-back working memory training, compared to a sham group (Ke et al., 2019). The study also reported near-transfer effects to a similar, untrained 3-back version of the task. Similarly, Weller et al. (2020) found that, compared to sham stimulation, anodal tDCS with a 1 mA stimulation intensity applied to the left PFC improved CCT performance gains. Another recent study by Sommer and Plewnia (2021) examined the required tDCS dosage but reported that the addition of tDCS only resulted in a small increase in CCT effectiveness, concluding that any tDCS effects were potentially overshadowed by a larger CCT effect. Segrave et al. (2014) found no immediate effects of tDCS after CCT, but did report decreased depressive symptomatology at 3 week follow-up. It is possible that the effects of CCT might be more pronounced long-term as opposed to immediately after training. In line with this, Hoorelbeke and Koster (2017) reported effects of CCT to be more strongly pronounced at 3 months follow-up than at post-training, suggesting that emotional transfer effects following CCT may gradually develop over time. Indeed, several studies have shown long-term effects of CCT, among which one study reporting reduced need for clinical care at 1 year follow-up in MDD patients (Siegle et al., 2014). Furthermore, Hoorelbeke et al. (2021) observed task-specific cognitive transfer following CCT at 1 year follow-up in RMD patients, in addition to reduced risk for recurrence of depression and lower expressed need for psychotherapeutic interventions during the follow-up period.

## Cost-effectiveness

In spite of the challenges mentioned above, CCT is an intervention that lends itself well to online dissemination and could be scalable to meet the needs of the large population vulnerable to depression. Despite previous research reporting cost-effectiveness of internet- and mobile-based interventions (IMIs) for both treatment and prevention of depression (Paganini et al., 2018), studies examining the cost-effectiveness of CCT specifically are largely missing. A study in a sample of post-stroke patients compared the combination of cognitive behavioral therapy (CBT) and occupational and movement therapy to a computerized cognitive training program as a control intervention and found no convincing cost-effective differences (van Eeden et al., 2015). Similarly, economic evaluations on IMIs have found that they were likely to be cost-effective when compared to TAU (Conejo-Cerón et al., 2021). With increasing research and clinical interest in CCT in the context of depression, more research in its cost-effectiveness is required, both as a stand-alone intervention, as well as a combination with other anti-depressant interventions.

Furthermore, cost-effectiveness could potentially be improved by employing a stratified care approach (i.e., identifying patients who benefit most from a specific intervention and providing them access to this treatment) instead of a stepped care approach (i.e., most patients first access only low-intensity treatments and patients who remain symptomatic later access more intensive interventions). Delgadillo et al. (2021) found that stratified care was efficacious and cost-effective when compared to stepped care for psychological treatment of depressive symptomatology. However, similar studies for CCT specifically have not yet been conducted, even though CCT shows high potential to be integrated within such a treatment approach. As such, further research into the cost-effectiveness of CCT provides an important opportunity to build toward implementation of CCT in clinical practice.

## Future directions

Currently, the use of CCT has not been widely employed as an intervention in the context of depression, and more research is required to justify and facilitate the introduction of CCT in clinical practice. In addition to the need for future research into the working mechanisms underlying effects of CCT, moderators of training effects, treatment augmentation, and cost-effectiveness, one important aspect that has currently not received much attention, is the optimal training dosage and method of administration. That is, as to date, the dose (i.e., the number of sessions) required for cognitive and emotional transfer effects to occur, as well as how this relates to the long-term sustainability of those effects remains to be tested. A recent meta-analysis by Launder et al. (2021) found a moderating effect of dose on overall cognition, with larger CCT doses being associated with greater effect size estimates, but the same effect was not statistically significant for depressive symptomatology. The authors refer to higher heterogeneity and imprecision within dose subgroups as possible explanations. Indeed, the absence of a dose-response relationship for CCT results in the use of variable doses in current research, potentially contributing to the heterogeneity in reported effects. The investigation of an optimal dose-response holds several key factors: the length of one training session, the number of sessions employed, and how frequently they are delivered. These three factors have seen a wide variability in scientific literature examining the effects of CCT on depressive symptomatology and often also depend on the training by which the CCT is operationalized. For example, recent adaptive PASAT studies have mostly used ten sessions of 15 min each with a maximum of one session a day (Van den Bergh et al., 2020; Vervaeke et al., 2020; Hoorelbeke et al., 2022). Recent studies using the n-back have generally used slightly longer training sessions of 20–30 min and a higher dose, around 20 sessions (e.g., Beloe and Derakshan, 2019; Zhang et al., 2019). The location where the training sessions take place is variable as well, with some studies inviting participants to a research or clinical facility, whereas others handle CCT as a training a participant can conduct at home, unsupervised. More research into these areas and how they relate to training progress and effectiveness is required for a broader picture of the effects of training dose and training administration on depressive symptomatology. Ideally, a dose-response relationship would be established using preregistered randomized controlled trials (RCT), where individuals randomized over groups receive a different amount of CCT sessions, examining emotional and cognitive transfer effects. Here, it also is conceivable that optimal dosage differs pending individual characteristics and the type of training task used.

Given the finding that for some at-risk groups emotional transfer effects may be short-lived (e.g., Hoorelbeke et al., 2022), another interesting area, closely linked to the effect of training dose, is the examination of the use of booster sessions in CCT. Similar to boosters in other clinical interventions, additional booster sessions may be beneficial in maintaining or increasing long-term effects of CCT. The effectiveness of booster sessions has been examined for psychotherapy and general cognitive training, with reported beneficial effects such as preventing loss of cognitive functioning (Aramaki and Yassuda, 2011; Felix et al., 2021) or maintained effectiveness of psychological treatment (Gearing et al., 2013; Wesner et al., 2015). However, it is currently unclear whether booster sessions increase the effectiveness of CCT in the context of depression. Equally unknown is *when* these booster sessions could prove beneficial, either at a fixed interval (e.g., after a fixed amount of time after completion of the last training session) or at an individualized, variable interval (e.g., when people are experiencing an increase in depressive symptomatology or related risk factors). One possible way to examine the effect of booster sessions would be *via* Experience Sampling Methodology (ESM), which allows to regularly assess functioning of individuals over time. Upon displaying increased depressive symptomatology or other early warning signs for recurrence of depression (e.g., Wichers and Groot, 2016), additional booster sessions could be administered. This type of design would allow to examine if the addition of booster sessions at crucial moments could result in more durable treatment effects.

(Remitted) depressed patients do not uniformly portrait cognitive impairments: whilst some people might have more trouble remembering information, others might complain about attentional deficits. Currently, CCT provides the same type of training for everyone, regardless of their individual cognitive impairments and needs. Future research should focus on examining specific types of cognitive impairments and could investigate if providing a personalized treatment might lead to more beneficial outcomes in both cognitive recovery, as well as decreased risk of recurrence.

## Conclusion

CCT has been gaining traction as a psychological intervention in the context of depression. Due to the advantages and wide-spread availability of modern technologies, individualized CCT could provide rapid and low-cost help. However, several key factors of how CCT can be effectively implemented are still unclear. Future research should focus on open questions, such as investigating a dose-response relationship, examining the efficacy and the optimal timing of booster sessions, as well as exploring the effectiveness of a combination of CCT with other frequently used interventions in the context of depression. Interindividual differences in (cognitive) needs could be considered when planning the use of CCT for ameliorating cognitive impairments and depressive symptomatology. Finally, the efficacy of a stratified medicine approach for CCT should be investigated, focusing on the previously mentioned key points, as well as taking baseline cognitive abilities into account, in order to provide a personalized treatment for depression, tailored to individual needs.

## Author contributions

YVZ, KH, and EK developed the framework of the review. All authors provided critical contributions and revisions.

## Conflict of interest

The authors declare that the research was conducted in the absence of any commercial or financial relationships that could be construed as a potential conflict of interest.

## Publisher’s note

All claims expressed in this article are solely those of the authors and do not necessarily represent those of their affiliated organizations, or those of the publisher, the editors and the reviewers. Any product that may be evaluated in this article, or claim that may be made by its manufacturer, is not guaranteed or endorsed by the publisher.
